# Supplementary material for: Freshwater sponges in the southeastern U.S. harbor unique microbiomes that are influenced by host and environmental factors
Source: PeerJ. 2025 Jan 30;13:e18807. doi: 10.7717/peerj.18807 (PMC11787800; doi:10.7717/peerj.18807)
Supplement: Supplemental Information 1 [file peerj-13-18807-s001.docx]

**Supplementary Information for:**

**Freshwater Sponges in the Southeastern U.S. Harbor Unique Microbiomes that are Influenced by Host and Environmental Factors**

**Jacqueline G. Keleher^1^, Taylor Strope^1,2^, Noah Estrada^1^, Allison M. Griggs Mathis^1^, Cole G. Easson^3^, Cara L. Fiore^1^**

**^1^Department of Biology, Appalachian State University, Boone, North Carolina, USA**

**^2^Department of Biochemistry and Molecular Biology, University of Kansas Medical Center, Kansas City, Kansas, USA**

**^3^Biology Department, Middle Tennessee State University, Murfreesboro, Tennessee, USA**

**Corresponding author:**

**Cara Fiore**

**fiorec@appstate.edu**

*16S rRNA gene cloning and sequencing methods and results*

Preliminary cloning and sequencing of 16S rRNA genes were conducted in 2016 on one *Radiospongilla crateriformis* collected (July 15, 2016) from the Watauga River (36.255353 N -81.850502 W), just downstream from where the rest of the sampling was conducted for this study in 2017. DNA was extracted using CTAB as described for the 2017 sponges in the main text and the V4 region of the 16S rRNA gene was amplified using the original forward and modified reverse earth microbiome primers (515f: GTGCCAGCMGCCGCGGTAA, 806r: GGACTACHVGGGTWTCTAAT; Caporaso et al., 2011). The thermal cycler protocol included an initial denaturation step at 94°C for 30 s, then 30 cycles of 98°C for 10 s, 50°C for 1 min, and 68°C for 1 min, followed by extension at 68°C for 5 min and hold at 4°C. The PCR setup included: Hot start *Taq* 2X Master Mix (New England BioLabs, Ipswich, MA, USA) following the manufacturer protocol for 50 µl volume reaction: 1 µl of 10 µM each primer, 25 ng of DNA, and nuclease-free water to 50 µl. The PCR product was cloned with *Escherichia coli* using 10-beta competent cell kit and pMiniT vector, which includes growth media (New England Biolabs, Ipswitch, MA, USA). Ten clones were selected for plasmid DNA extraction (PureYield Plasmid Miniprep system, Promega, Madison, WI, USA) and Sanger sequencing at Eurofins Genomics (Louisville, KY, USA) using the forward plasmid primer T7 (5’ -TAATACGACTCACTATAGGG). Sequencing results were analyzed as described in the main text.

Of the ten clones sequenced, three were most similar to the 16S rRNA gene of freshwater sponge mitochondria, and seven were similar to those of other uncultured bacteria. One sequence yielded similarity to the 16S rRNA gene of bacteria in the sponge, *Lubomirskia baicalensis*, of Lake Baikal (top seven BLAST matches) and this sequence was deposited in NCBI (accession PP930792). This percent identity of the sequence from *R. crateriformis* to those isolates from *L. baicalensis* (> 96% and 95% coverage) were higher than any free-living freshwater bacterial isolates from the United States (second highest similarity was 95% identity and 96% coverage to a Bacteroidetes isolate (HQ663172.1) from Lake Mendota, WI, USA).

*Sponge Identification by Taxonomic Marker Gene Analysis*

Sponge sample DNA was also used to amplify two sponge genes, the cytochrome oxidase 1 gene (COI) and the 18S rRNA with the internal transcribed spacer region (ITS) for taxonomic identification. For the COI gene, the primers LCO1490 (5'-GGTCAACAATCATAAAGATATTGG-3') and HC2198 (5'-TAAACTTCAGGGTGACCAAAAAATCA-3') were used to amplify a 708 bp region of the COI gene (Folmer et al., 1994). For 18S rRNA and ITS, the forward primer FW13 (5’-TACACACCGCCCGTCGCTACTA-3’) and reverse primer 1278 (5’-CTYYGACGTGCCTTTCCAGGT-3’) were used to amplify a region that includes the 3’ end of the 18S rRNA, ITS1, 5.8S rRNA, and 5’ end of 28S rRNA, yielding a 1060 bp product (Itskovich et al., 2008). PCR protocol followed the published protocols for the thermal cycler (Folmer et al., 1994; Itskovich et al., 2008). The PCR setup for both primer sets included: Hot start *Taq* 2X Master Mix (New England BioLabs, Ipswich, MA, USA) following the manufacturer protocol for 50 µl volume reaction: 1 µl of 10 µM each primer, 25 ng of DNA, and nuclease-free water to 50 µl. A portion (10 µl) of the resulting PCRs were used in gel electrophoresis (1% gel) to confirm the DNA band of the correct size. If a band of the expected size was present, these PCRs, along with the forward primer for each primer set, were sent to Eurofins Genomics for Sanger sequencing. The resulting chromatograms were viewed and manually inspected with 4Peaks v1.8 software (https://nucleobytes.com/4peaks/). The corresponding DNA sequences were saved as fasta files and were used with the search tool BLAST (Altschul et al., 1990) to compare against the NCBI non-redundant (nr) nucleotide database.

*Supplementary Figures*


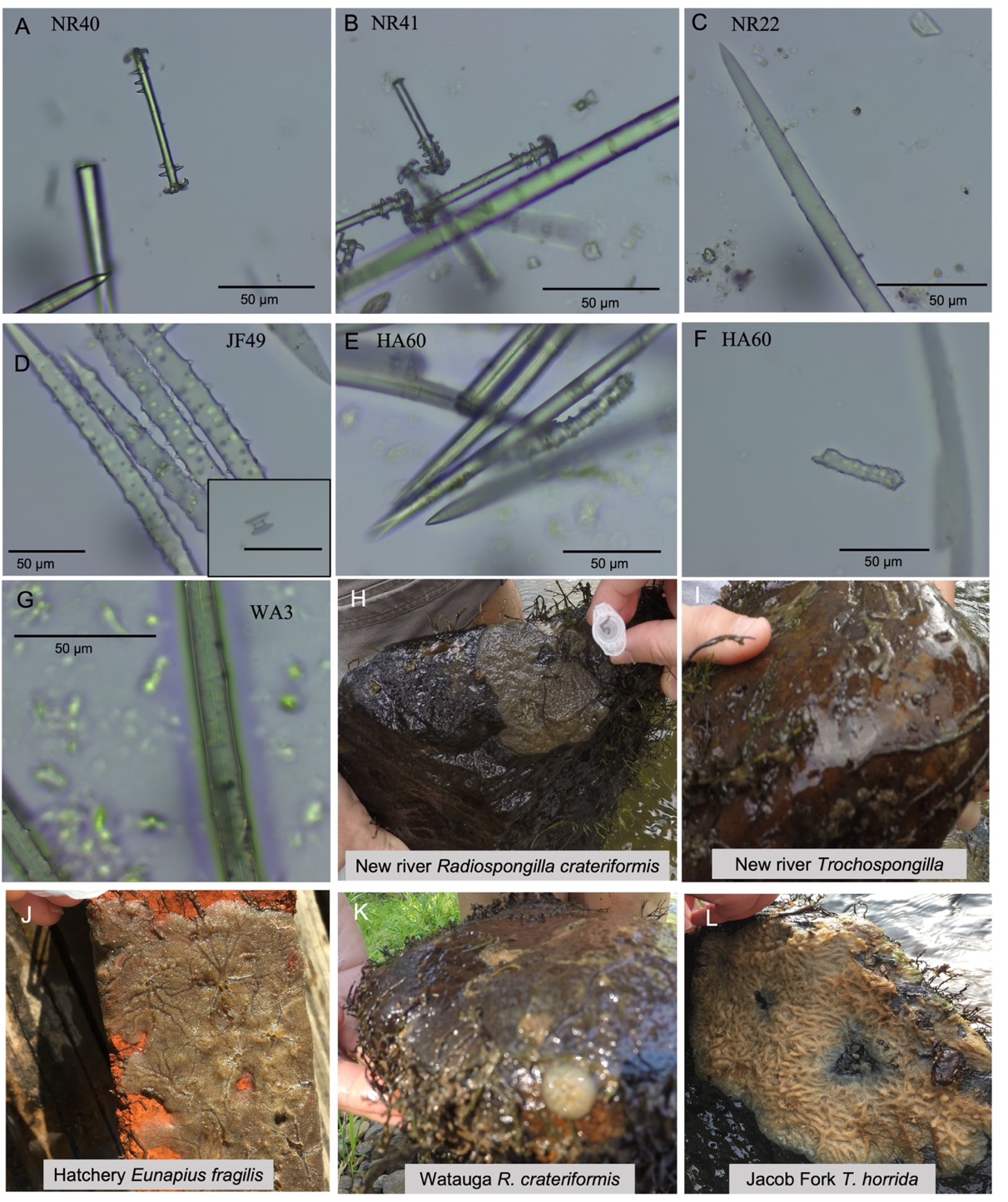


Figure S1. Subset of spicule and sponge images. Oxea megascleres and gemmulosceres of *Radiospongilla crateriformis* (A-C), oxea megasclere and gemmoscleres of *Trochospongilla horrida* (D), oxea megascleres and two gemmosclere types of *Eunapius fragilis* (E,F), oxea megascleres of putative *R. crateriformis* from the Watauga River (G), and example live sponge images of sampled specimens (H-L). Sample identification by site and/or number are shown for each image. NE = New River, HA = hatchery, JF = Jacob Fork River, WA = Watauga River.


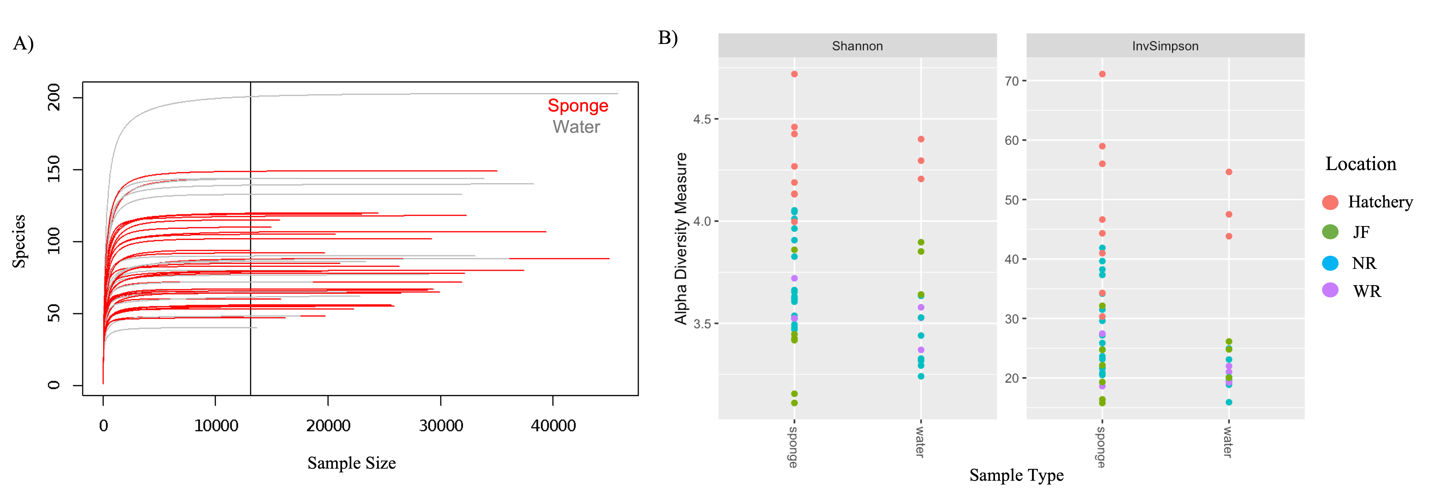


Figure S2. Rarefaction (A) and alpha diversity (B) of 16S rRNA gene sequencing of freshwater sponges and corresponding water samples. Sponge and water samples were sequenced to saturation (A). The vertical line marks the sample with the lowest number of reads. Shannon and inverse Simpson diversity metrics were used to examine alpha diversity in sponge and water samples, colors correspond to sample location (B).

Figure S3. Temperature data for cranberry creek (36.5697, -81.327), located near New River State Park, where the New River sponges were sampled each month July through September. Temperature data were obtained from the New River Conservancy (<https://www.newriverconservancy.org/wqhome>). The blue bars denote the year of sampling for the current study and temperature for other years are provided for context.


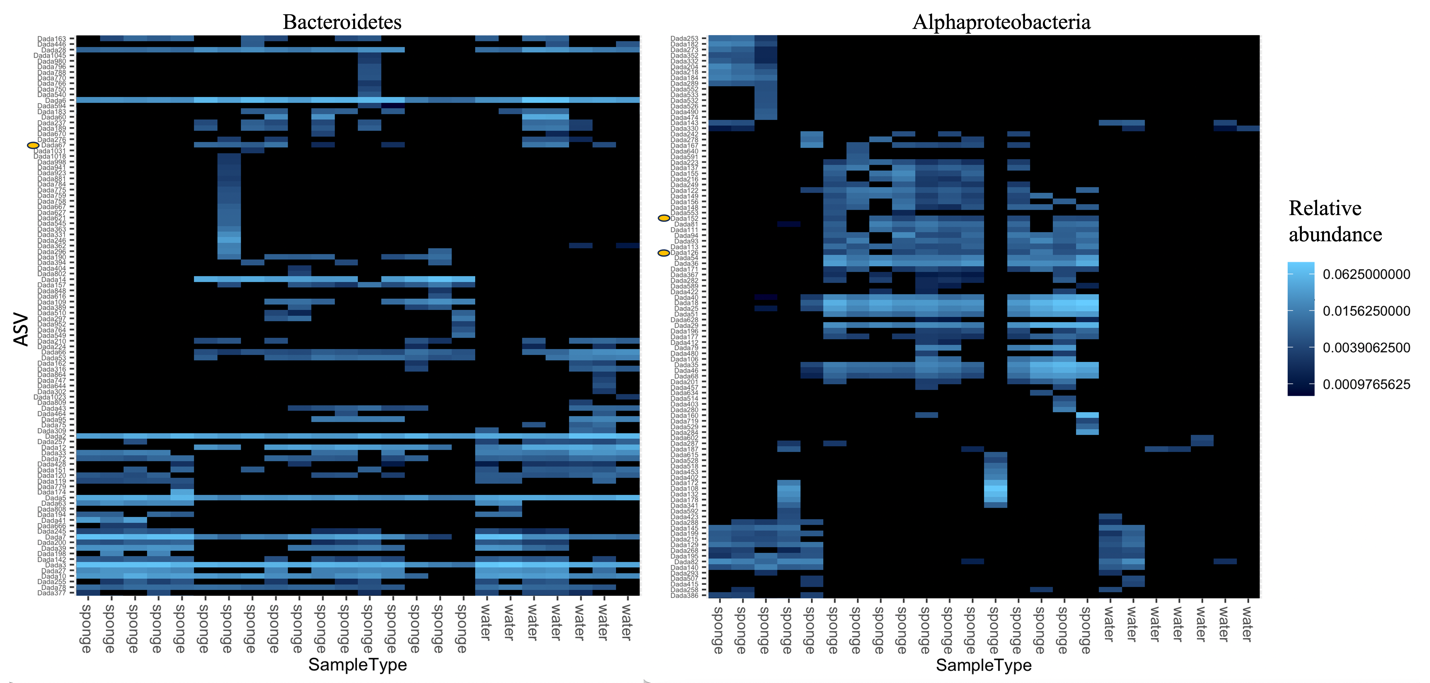


Figure S4. Heatmaps of the “top 100” ASVs for the taxonomic classes of Bacteroidetes (Bacteroidota) and Alphaproteobacteria (Proteobacteria). The orange markers denote taxa listed in Table 3.

Figure S5. Inorganic nutrients from the New River (NR) and Jacob Fork River (JF) sites in 2017 and 2018. Average with standard deviation shown for samples with multiple replicates. Note difference in y-axis for each nutrient.

**References**

Altschul SF, Gish W, Miller W, Myers EW, Lipman DJ. 1990. Basic local alignment search tool.

Journal of Molecular Biology 215(3):403–410 DOI 10.1016/S0022-2836(05)80360-2.

Caporaso JG, Lauber CL, Walters WA, Berg-Lyons D, Lozupone CA, Turnbaugh PJ, Fierer N, Knight R. 2011. Global patterns of 16S rRNA diversity at a depth of millions of sequences per sample. *Proceedings of the National Academy of Sciences* 108:4516–4522. DOI 10.1073/pnas.1000080107.

Durand, M. D., & Olson, R. J. 1996. Contributions of phytoplankton light scattering and cell concentration changes to diel variations in beam attenuation in the equatorial Pacific from flow cytometric measurements of pico-, ultra-and nanoplankton. *Deep Sea Research Part II: Topical Studies in Oceanography*, *43*(4-6), 891-906. DOI 10.1016/0967-0645(96)00020-3.

Folmer O, Black M, Hoeh W, Lutz R, Vrijenhoek R. 1994. DNA primers for amplification of

mitochondrial cytochrome c oxidase subunit I from diverse metazoan invertebrates. Molecular

Marine Biology and Biotechnology 3:294–299. PMID 7881515

Itskovich V, Gontcharov A, Masuda Y, Nohno T, Belikov S, Efremova S, Meixner M,

Janussen D. 2008. Ribosomal ITS sequences allow resolution of freshwater sponge phylogeny

with alignments guided by secondary structure prediction. Journal of Molecular Evolution

67(6):608–620 DOI 10.1007/s00239-008-9158-5.

Lomas, M. W., Steinberg, D. K., Dickey, T., Carlson, C. A., Nelson, N. B., Condon, R. H., & Bates, N. R. 2010. Increased ocean carbon export in the Sargasso Sea linked to climate variability is countered by its enhanced mesopelagic attenuation. *Biogeosciences*, *7*(1), 57-70. DOI 10.5194/bg-7-57-2010.

Marie, D., Simon, N., & Vaulot, D. 2005. Phytoplankton cell counting by flow cytometry. *Algal culturing techniques*, *1*, 253-267. DOI 10.1016/B978-012088426-1/50018-4.

Vaulot, D., Courties, C., & Partensky, F. 1989. A simple method to preserve oceanic phytoplankton for flow cytometric analyses. *Cytometry: The Journal of the International Society for Analytical Cytology*, *10*(5), 629-635. DOI 10.1002/cyto.990100519.
