# Supplementary material for: Freshwater sponges in the southeastern U.S. harbor unique microbiomes that are influenced by host and environmental factors
Source: PeerJ. 2025 Jan 30;13:e18807. doi: 10.7717/peerj.18807 (PMC11787800; doi:10.7717/peerj.18807)
Supplement: Supplemental Information 2 [file peerj-13-18807-s002.pdf]

Keleher et al. Submitted to PeerJ: Microbiome of Western NC Freshwater Sponges
NCBI Accessions Sponge 18S and COX1 – May 2024 to be released August 2024
Until accessible, sequences can be found below accession numbers:

COX1
SUB14488464 RSNR10_NR20 PP853474
SUB14488464 RSNR13_NR21 PP853475
SUB14488464 THJF23_JF52 PP853476
SUB14488464 THJF24_JF53 PP853477
SUB14488464 EFHa1_Hatch6 PP853478
SUB14488464 EFHa2_Hatch7 PP853479
SUB14488464 EFHa3_Hatch8 PP853480
SUB14488464 EFHa4_Hatch9 PP853481

18S and ITS
SUB14491266 NR5_CF29 PP853638
SUB14491266 NR6_CF30 PP853639
SUB14491266 NR17_CF24 PP853640
SUB14491266 JF21_CF50 PP853641
SUB14491266 JF22_CF51 PP853642
SUB14491266 Ha4b_CF9 PP853643
SUB14491266 Ha6_CF59 PP853644
SUB14491266 Ha7_CF60 PP853645

16S clone:
We have provided GenBank accession number(s) for your nucleotide sequence(s):

SUB14491334 RSclone_5f_NR PP930792

COX1:
>RSNR10_NR20 Radiospongilla crateriformis Cytochrome oxidase subunit 1 gene, partial cds; mitochondrial
TGTTAGGGGATGATCAATTATATAATGTTATAGTTACAGCCCATGCTTTTCTAATGATATTTTTCTTAGTTATGC
CAGTAATGATTGGGGGATTTGGAAATTGATTCGTGCCATTATATATTGGTGCACCCGATATGGCTTTTCCAA
GATTAAACAATATTAGTTTTTGATTATTACCTCCGGCTTTAACTCTATTATTAGGATCTGCTTTTGTAGAGCAA
GGGGTTGGTACAGGATGGACAGTATATCCCCCTTTAGCAGGCATACAAGCGCATTCTGGGGGATCGGTTG
ATATGGCAATATTTAGTCTTCACTTGGCGGGTATTTCTTCGATATTAGGGGCTATGAATTTTATCACAACAATC
TTTAATATGAGAGCGCCCGGTATTACAATGGATAGACTGCCATTATTTGTATGATCTATTTTAATAACAGCCTT
TTTATTATTATTATCTTTACCTGTATTAGCTGGTGGTATAACAATGCTTTTAACAGATAGAAATTTTAATACAAC
ATTCTTTGATCCTGCTGGAGGAGGAGACCCAATACTATTTCAACATTTATTTTGATTTTTTGGTCACCC
>RSNR13_NR21 Radiospongilla crateriformis Cytochrome oxidase subunit 1 gene, partial cds; mitochondrial
CCCATGCTTTTCTAATGATATTTTTCTTAGTTATGCCAGTAATGATTGGGGGATTTGGAAATTGATTCGTGCC
ATTATATATTGGTGCACCCGATATGGCTTTTCCAAGATTAAACAATATTAGTTTTTGATTATTACCTCCGGCTTT
AACTCTATTATTAGGATCTGCTTTTGTAGAGCAAGGGGTTGGTACAGGATGGACAGTATATCCCCCTTTAGC
AGGCATACAAGCGCATTCTGGGGGATCGGTTGATATGGCAATATTTAGTCTTCACTTGGCGGGTATTTCTTC
GATATTAGGGGCTATGAATTTTATCACAACAATCTTTAATATGAGAGCGCCCGGTATTACAATGGATAGACTG
CCATTATTTGTATGATCTATTTTAATAACAGCCTTTTTATTATTATTATCTTTACCTGTATTAGCTGGTGGTATAA
CAATGCTTTTAACAGATAGAAATTTTAATACAACATTCTTTGATCCTGCTGGAGGAGGAGACCCAATACTAT
TTCAACATTTATTTTGATTTTTTGGTCACCC
>THJF23_JF52 Trochospongilla horrida Cytochrome oxidase subunit 1 gene, partial cds; mitochondrial
TGTTAGGGGATGATCAATTATATAATGTTATAGTTACAGCCCATGCTTTTCTAATGATATTTTTCTTAGTTATGC
CAGTAATGATTGGGGGATTTGGAAATTGATTCGTGCCATTATATATTGGTGCACCCGATATGGCTTTTCCAA
GATTAAACAATATTAGTTTTTGATTATTACCTCCGGCTTTAACTCTATTATTAGGATCTGCTTTTGTAGAGCAA
GGGGTTGGTACCGGATGGACAGTATACCCCCCTTTAGCAGGCATACAAGCACATTCTGGGGGATCAGTTG
ATATGGCAATATTTAGTCTTCACTTGGCGGGTATTTCTTCGATATTAGGGGCTATGAATTTTATCACAACAATC
TTTAATATGAGAGCGCCTGGTATTACAATGGATAGAATGCCATTATTTGTATGATCTATTTTAATAACAGCCTT
TTTATTATTATTATCTTTACCTGTATTAGCTGGTGGTATAACAATGCTTTTAACAGATAGAAATTTTAATACAAC
ATTCTTTGATCCTGCTGGGGGAGGAGACCCAATACTATTTCAACATTTATTTTGATTTTTTGGTCAC
>THJF24_JF53 Trochospongilla horrida Cytochrome oxidase subunit 1 gene, partial cds; mitochondrial
CCATGCTTTTCTAATGATATTTTTCTTAGTTATGCCAGTAATGATTGGGGGATTTGGAAATTGATTCGTGCCAT
TATATATTGGTGCACCCGATATGGCTTTTCCAAGATTAAACAATATTAGTTTTTGATTATTACCTCCGGCTTTA
ACTCTATTATTAGGATCTGCTTTTGTAGAGCAAGGGGTTGGTACAGGATGGACAGTATATCCCCCTTTAGCA
GGCATACAAGCGCATTCTGGGGGATCGGTTGATATGGCAATATTTAGTCTTCACTTGGCGGGTATTTCTTCG
ATATTAGGGGCTATGAATTTTATCACAACAATCTTTAATATGAGAGCGCCCGGTATTACAATGGATAGACTGC
CATTATTTGTATGATCTATTTTAATAACAGCCTTTTTATTATTATTATCTTTACCTGTATTAGCTGGTGGTATAAC
AATGCTTTTAACAGATAGAAATTTTAATACAACATTCTTTGATCCTGCTGGAGGAGGAGACCCAATACTATTT
CAACATTTATTTTGATTTTTTGGTCACCCTGAAG
>EFHa1_Hatch6 Eunapius fragilis Cytochrome oxidase subunit 1 gene, partial cds; mitochondrial
CCATGCTTTTCTAATGATATTTTTCTTAGTTATGCCAGTAATGATTGGGGGATTTGGAAATTGATTCGTGCCAT
TATATATTGGTGCACCCGATATGGCTTTTCCAAGATTAAACAATATTAGTTTTTGATTATTACCTCCGGCTTTA

ACTCTATTATTAGGATCTGCTTTTGTAGAGCAAGGGGTTGGTACAGGATGGACAGTATATCCCCCTTTAGCA
GGCATACAAGCGCATTCTGGGGGATCGGTTGATATGGCAATATTTAGTCTTCACTTGGCGGGTATTTCTTCG
ATATTAGGGGCTATGAATTTTATCACAACAATCTTTAATATGAGAGCGCCCGGTATTACAATGGATAGACTGC
CATTATTTGTATGATCTATTTTAATAACAGCCTTTTTATTATTATTATCTTTACCTGTATTAGCTGGTGGTATAAC
AATGCTTTTAACAGATAGAAATTTTAATACAACATTCTTTGATCCTGCTGGAGGAGGAGACCCAATACTATTT
CAACATTTATTTTGATTTTTTGGTCACCCTGAAGTTT
>EFHa2_Hatch7 Eunapius fragilis Cytochrome oxidase subunit 1 gene, partial cds; mitochondrial
CCATGCTTTTCTAATGATATTTTTCTTAGTTATGCCAGTAATGATTGGGGGATTTGGAAATTGATTCGTGCCAT
TATATATTGGTGCACCCGATATGGCTTTTCCAAGATTAAACAATATTAGTTTTTGATTATTACCTCCGGCTTTA
ACTCTATTATTAGGATCTGCTTTTGTAGAGCAAGGGGTTGGTACAGGATGGACAGTATATCCACCTTTAGCA
GGTATACAAGCACATTCTGGGGGATCAGTTGATATGGCAATATTTAGTCTTCACTTGGCGGGTATTTCTTCG
ATATTAGGGGCTATGAATTTTATCACAACAATCTTTAATATGAGAGCGCCCGGTATTACAATGGATAGAATGC
CATTATTTGTATGATCTATTTTAATAACAGCCTTTTTATTATTATTATCTTTACCTGTATTAGCTGGTGGTATAAC
AATGCTTTTAACAGATAGAAATTTTAATACAACATTCTTTGATCCTGCTGGAGGAGGAGACCCTATACTATTT
CAACATTTATTTTGATTTTTTGGTCACCCTGAAGTT
>EFHa3_Hatch8 Eunapius fragilis Cytochrome oxidase subunit 1 gene, partial cds; mitochondrial
CCCATGCTTTTCTAATGATATTTTTCTTAGTTATGCCAGTAATGATTGGGGGATTTGGAAATTGATTCGTGCC
ACTATATATTGGTGCACCCGATATGGCTTTTCCAAGATTAAATAATATTAGTTTTTGATTATTACCTCCGGCTTT
AACTCTATTATTAGGATCTGCTTTTGTAGAGCAAGGGGTTGGTACAGGATGGACAGTATATCCCCCTTTAGC
AGGGATACAAGCACATTCTGGGGGATCAGTTGATATGGCAATATTTAGTCTTCACTTGGCGGGTATTTCTTC
GATATTAGGGGCTATGAATTTTATCACTACAATCTTTAATATGAGAGCGCCCGGTATTACAATGGATAGAATG
CCATTATTTGTATGATCTATTTTAATAACAGCCTTTTTATTATTATTATCTTTACCTGTATTAGCTGGTGGTATAA
CAATGCTTTTAACAGATAGAAATTTTAATACAACATTTTTTGATCCTGCTGGAGGAGGAGACCCAATACTAT
TTCAACATTTATTTTGATTTTTTGGTCACCCTGAAGTTTA
>EFHa4_Hatch9 Eunapius fragilis Cytochrome oxidase subunit 1 gene, partial cds; mitochondrial
CAGCCCATGCTTTTCTAATGATATTTTTCTTAGTTATGCCAGTAATGATTGGGGGATTTGGAAATTGATTCGT
GCCACTATATATTGGTGCACCCGATATGGCTTTTCCAAGATTAAATAATATTAGTTTTTGATTATTACCTCCGG
CTTTAACTCTATTATTAGGATCTGCTTTTGTAGAGCAAGGGGTTGGTACAGGATGGACAGTATATCCCCCTTT
AGCAGGGATACAAGCACATTCTGGGGGATCAGTTGATATGGCAATATTTAGTCTTCACTTGGCGGGTATTTC
TTCGATATTAGGGGCTATGAATTTTATCACTACAATCTTTAATATGAGAGCGCCCGGTATTACAATGGATAGA
ATGCCATTATTTGTATGATCTATTTTAATAACAGCCTTTTTATTATTATTATCTTTACCTGTATTAGCTGGTGGTA
TAACAATGCTTTTAACAGATAGAAATTTTAATACAACATTTTTTGATCCTGCTGGAGGAGGAGACCCAATAC
TATTTCAACATTTATTTTGATTTTTTGGTCACC

18S and ITS:
>NR5_CF29 Radiospongilla crateriformis 18S ribosomal RNA gene, partial, internal transcribed spacer 1, complete; 5.8S ribosomal RNA gene, complete; internal transcribed spacer 2, partial
GAACCTGCGGAAGGATCATTACCGTACCATTTCGGGGGATTCGTTTTCCCTGGATCCACTGTGCACTAGGC
TGCGGCCCGCCAGGAGCCGCGTTTGGGGAGGTCGCCCTCGGGTGCGAGGTCGACGCGAGTCGGCGAGA
GAGATCCCTCTGCCGGCCTCTCCGACGCGGTTCCAGGGCCGCAGTTCTTATTATTTTTTTTCTCACGAACCG
TGGAGTTACCAACGGGTCTAGTTTCGTTTCGTTTGTCGAGGAGACAACGTAAAAAAGTTGAGACAACTTC
TAACGGTGGACCCCTCGGCTCGTGCGTCGATGAAGAACGCAGCAAACTGCGATACGTAGTGTGAATTGCA
GAATTCCGTGAATCATCGAGTCTTTGAACGCAAATTGCGCCCTCGGTTTGAAGCCGGGGGCACGTCTGTCT
GAGCGTCCGTTTCGTTTCTTGTCTCCCCGGCGAGCGTTTCTCCCAAAAAGAGGAACGTTTTCGCTCCGGC
GGTTGGCGTGTTGAGGCGTCGTCCGGCGACGGGAGTCCCTTGAAGTGCGAAGCGCTCCGGATCGAAGG
ACTCGGATCAGCTCGAGTGCCCTTCCACCTTGCGCGT
>NR6_CF30 Radiospongilla crateriformis 18S ribosomal RNA gene, partial, internal transcribed spacer 1, complete; 5.8S ribosomal RNA gene, complete; internal transcribed spacer 2, partial
CCTGCGGAAGGATCATTACCGTACCATTTCGGGGGATTCGTTTTCCCTGGATCCACTGTGCACTAGGCTGC
GGCCCGCCGGAGCCGCGTTTGGGGAGGTCGCCCTCGGGTGCGAGGTCGACGCGAGTCGGCGAGAGAG
ATCCCTCCGCCGGCCTCTCCGACGCGGTTCCAGGGCCGCAGTTCTTATAATTTTTTTTCTCACGAACCGTGG
AGTTACCAACGGGTCTAGTTTCGTTTCGTTTGTCGAGGAGACAACGTAAAAAAGTTGAGACAACTTCTAAC
GGTGGACCCCTCGGCTCGTGCGTCGATGAAGAACGCAGCAAACTGCGATACGTAGTGTGAATTGCAGAAT
TCCGTGATTCATCTAGTCTTTGAACGCTAATTGCGCCCTCTGTTTGAAGCCGGGGGCACGTCTGTCTGAGC
GTCCGTTTCGTTTCTGTCTCCCCGGCGAGCGTTTCTCCCAAAAAGAGGAACGTTTTTTGCTCCGGCGGATG
GCGTGTTGAGGCGTCATCCGGCAACAG
>NR8_CF18 Radiospongilla crateriformis 18S ribosomal RNA gene, partial; internal transcribed spacer 1, partial
GAACCTGCGGAAGGATCATTACCGTACAATTTCGGGGATTTTCTTCCCTGGATCCACTGTGCACCAGGCTG
CGGCCCGCTATGCCGCGTTTTGGGGAGGTATGGCCCTCGGGCCGTCCTCCCCGACGCGGTGGGCCGCAG
TTCTTAAACTTTATTTTCGAACTCGAGCGGACCGAACGGGTATCTTTTTTTT
>NR17_CF24 Radiospongilla crateriformis 18S ribosomal RNA gene, partial, internal transcribed spacer 1, complete; 5.8S ribosomal RNA gene, complete; internal transcribed spacer 2, complete; 28S ribosomal RNA gene, partial
ATTTAGAGGAAGTAAAAGTCGTAACAAGGTTTCCGTAGGTGAACCTGCGGAAGGATCATTACCGTACCGTT
TCGGGGAATTCGTTCCCTCGATCCACTGTGCACTAGGCTGCGGCCCGCCAAAGCCGCGTCGGGGAGGTCG
TTCTCGGGTGCGAGGACGACGCGAGTCGGCGCGAGAGAACCCTCCGCCGGCCTCTCCGACGCGGAGGG
CCGCAGTTCTCGCTCTTTTTTTTCTCGAACCGTAGCGTACCGAACGGGTTGCTGTTTTTATCGTATTGTCGA
GGAGACAACGTATAAAAGTTTGAGACAACTTCTAACGGTGGACCCCTCGGCTCGTGCGTCGATGAAGAAC
GCAGCAAACTGCGATACGTAGTGTGAATTGCAGAATTCCGTGAATCATCGAGTCTTTGAACGCAAATTGCG
CCCTCGGTTCGAAGCCGGGGGCACGTCTGTCTGAGCGTCCGTTTCCTTTTGGCGTCTCCGCGTCGGGCGG
TCTTCCGACCGACCGGGAGGCGCGTCGTTGAGGCGTCGTCCGAAATTCGGGCGTCCCTTGAAGAGCGAA
GCGCTCCGGTTCGAAGGGCAAACCTCCTCTCGAGTGCCCTTCCACCTTGCGCGTCGGGAACTCGACGATG
ACAAGGGGAAGGGCGCCTCGTCTGCGAGGACCTGCGTTCCAGAGCCGCACCGAAAGCGGATTTATCGCG
GGAGAGCTCGTCTCTCCTCCATCCTGGACCTCAGCTCAGGCGTGACTACCCGCTGAATTTAAGCATATCAAT
AAGCGGAGGAAAAGAAACCAACAGGGATTCCCCCAGTAACGGCGAGCGAAGCGGGAATAGCTCGAGCC
TTAAATCTCCGGCGCACAGCCGGCGAATTGTAGCCGAGAGAGGCACCTGCGCTCGGCAGGCGGTCGACC
AAAGTTGACCTGGAAG

>JF21_CF50 Trochospongilla horrida 18S ribosomal RNA gene, partial, internal transcribed spacer 1, complete; 5.8S ribosomal RNA gene, complete; internal transcribed spacer 2, complete; 28S ribosomal RNA gene, partial
GGTGAACCTGCGGAAGGATCATTACCGTACCGTTTCGGGGAATTCGTTCCCTCGATCCACTGTGCACTAGG
CTGCGGCCCGCCAAAGCCGCGTCGGGGAGGTCGTTCTCGGGTGCGAGGACGACGCGAGTCGGCGCGAG
AGAACCCTCCGCCGGCCTCTCCGACGCGGAGGGCCGCAGTTCTCGCTCTTTTTTTTCTCGAACCGTAGCGT
ACCGAACGGGTTGCTGTTTTTATCGTATTGTCGAGGAGACAACGTATAAAAGTTTGAGACAACTTCTAACG
GTGGACCCCTCGGCTCGTGCGTCGATGAAGAACGCAGCAAACTGCGATACGTAGTGTGAATTGCAGAATT
CCGTGAATCATCGAGTCTTTGAACGCAAATTGCGCCCTCGGTTCGAAGCCGGGGGCACGTCTGTCTGAGC
GTCCGTTTCCTTTTGGCGTCTCCGCGTCGGGCGGTCTTCCGACCGACCGGGAGGCGCGTCGTTGAGGCGT
CGTCCGAAATTCGGGCGTCCCTTGAAGAGCGAAGCGCTCCGGTTCGAAGGGCAAACCTCCTCTCGAGTG
CCCTTCCACCTTGCGCGTCGGGAACTCGACGATGACAAGGGGAAGGGCGCCTCGTCTGCGAGGACCTGC
GTTCCAGAGCCGCACCGAAAGCGGTTTTATCGCGGGAGAGCTCGTCTCTCCTCCATCCTGGACCTCAGCTC
AGGCGTGACTACCCGCTGAATTTAAGCATATCAATAAGCGGAGGAAAAGAAACCAACAGGGATTCCCCCA
GTAACG
>JF22_CF51 Trochospongilla horrida 18S ribosomal RNA gene, partial, internal transcribed spacer 1, complete; 5.8S ribosomal RNA gene, complete; internal transcribed spacer 2, complete; 28S ribosomal RNA gene, partial
GTGAACCTGCGGAAGGATCATTACCGTACCATTTCGGGGGATTCGTTTTCCCTGGATCCACTGTGCACTAG
GCTGCGGCCCGCCAGGAGCCGCGTTTGGGGAGGTCGCCCTCGGGTGCGAGGTCGACGCGAGTCGGCGA
GAGAGATCCCTCTGCCGGCCTCTCCGACGCGGTTCCAGGGCCGCAGTTCTTATTATTTTTTTTCTCACGAAC
CGTGGAGTTACCAACGGGTCTAGTTTCGTTTCGTTTGTCGAGGAGACAACGTAAAAAAGTTGAGACAACT
TCTAACGGTGGACCCCTCGGCTCGTGCGTCGATGAAGAACGCAGCAAACTGCGATACGTAGTGTGAATTG
CAGAATTCCGTGAATCATCGAGTCTTTGAACGCAAATTGCGCCCTCGGTTTGAAGCCGGGGGCACGTCTG
TCTGAGCGTCCGTTTCGTTTCTTGTCTCCCCGGCGAGCGTTTCTCCCAAAAAGAGGAACGTTTTCGCTCCG
GCGGTTGGCGTGTTGAGGCGTCGTCCGGCGACGGGCGTCCCTTGAAGTGCGAAGCGCTCCGGTTCGAAG
GACTCGGCTCAGCTCGAGTGCCCTTCCACCTTGCGCGTCGGGAACTCGACGATGACAAGAGGGGAGAGG
CCTCGTTCGCGAGGTGATCCGGCGTACCAGAGCCAACACAGGTTTTTACACGAGGAGCTTTTTCACGAG
AGCTCTTCCATCCTGGACCTCAGCTCAGGCGTGACTACCCGCTGAATTTAAGCATATCAATAAGCGGAGGA
AAAGAAACCAACAGGGATTCCCCCAGTAACGGCGAGCGAAGCGGGAATAGCTCGAGCCTTAAATCTCCG
GCGCACAGCCGGCGAATTGTAGCCGAAAGAGGCACCTGCGCTCGGCAGGCGGTCGACCAAAGTTGACCT
GGAAAG
>JF23_CF52 18S Trochospongilla horrida 18S ribosomal RNA gene, partial
ACCTGCGGAAGGATCATTACCGTCTGTTCTCGGGGATCTCGATCCCTGCGATCCACCGTGCACTAGGCTGC
GGCCCGCCACGCCGCGTCGGGGAGGTCGCCCTCGGGTCAAGAGGGTGACGCAAGTCGGCGAGAGAGAT
CCCCTCGCCGGCCTCCCCGTCGCGGAGGGCCGCAGTTCTCTATTCCATTTTTTTTTCCAACCCTGGAATACC
CCCACCGGGTGGCCTTCCGGTTCCCTTTCCTGGCA
>Ha4b_CF9 18S Eunapius fragilis 18S ribosomal RNA gene, partial, internal transcribed spacer 1, complete; 5.8S ribosomal RNA gene, complete; internal transcribed spacer 2, complete; 28S ribosomal RNA gene, partial
CCTGCGGAAGGATCATTACCGTACCGTTTCGGGGAATTCGTTCCCTCGATCCACTGTGCACTAGGCTGCGG
CCCGCCAAAGCCGCGTCGGGGAGGTCGTTCTCGGGTGCGAGGACGACGCGAGTCGGCGCGAGAGAACC
CTCCGCCGGCCTCTCCGACGCGGAGGGCCGCAGTTCTCGCTCTTTTTTTTCTCGAACCGTAGCGTACCGAA
CGGGTTGCTGTTTTTATCGTATTGTCGAGGAGACAACGTATAAAAGTTTGAGACAACTTCTAACGGTGGAC

CCCTCGGCTCGTGCGTCGATGAAGAACGCAGCAAACTGCGATACGTAGTGTGAATTGCAGAATTCCGTGA
ATCATCGAGTCTTTGAACGCAAATTGCGCCCTCGGTTCGAAGCCGGGGGCACGTCTGTCTGAGCGTCCGT
TTCCTTTTGGCGTCTCCGCGTCGGGCGGTCTTCCGACCGACCGGGAGGCGCGTCGTTGAGGCGTCGTCCG
AAATTCGGGCGTCCCTTGAAGAGCGAAGCGCTCCGGTTCGAAGGGCAAACCTCCTCTCGAGTGCCCTTCC
ACCTTGCGCGTCAGGAACTCGACGATGACAAGGGGAAGGGCGCCTCGTCTGCGAGGACCTGCGTTCCAG
AGCCGCACCGAAAGCGGTGTTATCGCGGGAGAGCTCGTCTCTCCTCCATCCTGGACCTCAGCTCAGGCGT
GACTACCCGCTGAATTTAAGCATATCAATAAGCGGAGGAAGAGAAACCAACAGGGATTCCCCCAGTAACG
GCGAGCGAAGCGGGAATAGCTCCGAGCCTTAAATCTTCGGGCGCACAGCCGGCGAATTGTAACCGAAAA
AGGAACTGCNTTCGTCAGGCGGNGAACAAAGTGACCTGGAAGGGACGTTAAAG
>Ha6_CF59 18S Eunapius fragilis 18S ribosomal RNA gene, partial, internal transcribed spacer 1, complete; 5.8S ribosomal RNA gene, complete; internal transcribed spacer 2, complete; 28S ribosomal RNA gene, partial
ACCTGCGGAAGGATCATTACCGTACCATTTCGGGGGATTCGTTTTCCCTGGATCCACTGTGCACTAGGCTG
CGGCCCGCCAGGAGCCGCGTTTGGGGAGGTCGCCCTCGGGTGCGAGGTCGACGCGAGTCGGCGAGAG
AGATCCCTCTGCCGGCCTCTCCGACGCGGTTCCAGGGCCGCAGTTCTTATTATTTTTTTCTCACGAACCGT
GGAGTTACCAACGGGTCTAGTTTCGTTTCGTTTGTCGAGGAGACAACGTAAAAAAGTTGAGACAACTTCT
AACGGTGGACCCCTCGGCTCGTGCGTCGATGAAGAACGCAGCAAACTGCGATACGTAGTGTGAATTGCAG
AATTCCGTGAATCATCGAGTCTTTGAACGCAAATTGCGCCCTCGGTTTGAAGCCGGGGGCACGTCTGTCTG
AGCGTCCGTTTCGTTTCTTGTCTCCCCGGCGAGCGTTTCTCCCAAAAAGAGGAACGTTTTCGCTCCGGCG
GTTGGCGTGTTGAGGCGTCGTCCGGCGACGGGCGTCCCTTGAAGTGCGAAGCGCTCCGGTTCGAAGGAC
TCGGCTCAGCTCGAGTGCCCTTCCACCTTGCGCGTCGGGAACTCGACGATGACAAGAGGGGAGAGGCCT
CGTTCGCGAGGTGATCCGGCGTACCAGAGCCAACACAGGTTTTTTACACGAGGAGCTTTTTCACGAGAGC
TCTTCCATCCTGGACCTCAGCTCAGGCGTGACTACCCGCTGAATTTAAGCATATCAATAAGCGGAGGAAAA
GAAACCAACAGGGATTCCCCCAGTAACGGCGAGCGAAGCGGGAATAGCTCGAGCCTTAAATCTCCGGCG
CACAGCCGGCGAATTGTAGCCGAGAGAGGCACCTGCGCTCGGCAGGCGGTCGACCAAAGTTGACCTGG
AAA
>Ha7_CF60 Eunapius fragilis 18S ribosomal RNA gene, partial, internal transcribed spacer 1, complete; 5.8S ribosomal RNA gene, partial
TGAACCTGCGGAAGGATCATTACCGTCTGTTCTCGGGGATCTCGATCCCTGCGATCCACCGTGCACTAGGC
TGCGGCCCGCCACGCCGCGTCGGGGAGGTCGCCCTCGGGTCAAGAGGGTGACGCAAGTCGGCGAGAGA
GATCCCCTCGCCGGCCTCCCCGTCGCGGAGGGCCGCAGTTCTCTATTCCATTTTTTTTTCCAACCTTGGAGT
ACCCCGACAGGGTGGCCTTCGGTTTCTCTTTCTTGGCAAAAAAAAAACCAAAAAAACGAAAAACAACTTC
TAACGGTGGACCCCTCCGCTCGTGGGTCCATGAAGAACCCAACAAACTGGGATACGTAATGGGAATTGCA
AAATTCCGTGAATCATCGAGTCTTTGAACCCAAATTGGGCCCTCGGGTTGAAACCGGGGGCACCTCTGGC
TGAACGTC

16S rRNA gene from cloning:
>RSclone_5f_NR Uncultured bacterium sponge associated bacterium from Radiospongilla crateriformis 16S ribosomal gene, partial
GTCAGCCGGACTACCGGGGTATCTAATCCTGTTTGATCCCCACGCTTTCGTGCCTCNGCGTCAATATTTGCG
TAGCAAGCTGCCTTCNCAATTGGTGTTCTATGTCATATCNAAGCATTTCACCGCTACATGACATATNCCGCTT
ACCTCCACAATATTCAAGACAAATAGTATCAATGGCAGTTCTGAAGTTAAGCTTCAGGATTTCACCACTGAC
TTNNTTGCCCNCCTACGCACCCTTNAAANCCAGTGAATCCGGATAACGCTTGCACCCTCCGTATTACCGCG
GCTGCTGGCACA
